# Confocal Imaging and 3D Reconstruction to Determine How Genetic Perturbations Impact Presynaptic Morphology at the Mouse Calyx of Held

**DOI:** 10.21769/BioProtoc.4799

**Published:** 2023-09-05

**Authors:** Christian Keine, Tamara Radulovic, Mohammed Al-Yaari, Samuel M. Young

**Affiliations:** 1 Department of Anatomy and Cell Biology, University of Iowa, Iowa City, IA, USA; 2 Department of Human Medicine, University of Oldenburg, Oldenburg, Germany; 3 Research Center Neurosensory Science, Oldenburg, Germany; 4 Department of Otolaryngology, Iowa Neuroscience Institute, University of Iowa, Iowa City, IA, USA

**Keywords:** Calyx of Held, Presynaptic terminal, Confocal imaging, Three-dimensional reconstruction, Transcardial perfusion, Synaptic morphology

## Abstract

Neurons communicate via synapses—specialized structures that consist of a presynaptic terminal of one neuron and a postsynaptic terminal of another. As knowledge is emerging that mutations in molecules that regulate synaptic function underpin many neurological disorders, it is crucial to elucidate the molecular mechanisms regulating synaptic function to understand synaptic strength, plasticity, modulation, and pathology, which ultimately impact neuronal circuit output and behavior. The presynaptic calyx of Held is a large glutamatergic presynaptic terminal in the auditory brainstem, which due to its accessibility and the possibility to selectively perform molecular perturbations on it, is an ideal model to study the role of presynaptic proteins in regulating synaptic function. In this protocol, we describe the use of confocal imaging and three-dimensional reconstruction of the calyx of Held to assess alterations in gross morphology following molecular perturbation. Using viral-vector delivery to perform molecular perturbations at distinct developmental time points, we provide a fast and cost-effective method to investigate how presynaptic proteins regulate gross morphology such as surface area and synapse volume throughout the lifetime of a neuronal circuit.

Key features

Confocal imaging and 3D reconstruction of presynaptic terminals.

Used with a virus-mediated expression of mEGFP to achieve efficient, cell-type specific labeling of the presynaptic compartment.

Protocol was developed with the calyx of Held but is suitable for pre- and postsynaptic compartments of various neurons across multiple mammalian and invertebrate species.

## Background

The brain consists of billions of synapses, and many synaptic molecules have multiple roles regulating synapse morphology and synaptic transmission to control brain function (Shen and Cowan, 2010; Rosenberg et al., 2014). Therefore, combining physiological and imaging experiments provides powerful insight into the complex relationship between cellular structure and function (Marrone and Petit, 2002; Rollenhagen and Lübke, 2006; Wichmann and Moser, 2015; Sierksma et al., 2020). Genetic perturbations with viral-vector approaches allow for the targeted expression of different synaptic proteins in the pre- or postsynaptic cell in combination with a fluorescent reporter. Since the fluorescent reporter can readily be identified using light microscopy, this enables the use of laser scanning confocal microscopy in conjunction with the quantification of fluorescent signals in three-dimensional image stacks to examine the gross morphology of specific pre- or postsynaptic compartments at a fine scale. Here, we describe the use of confocal imaging of the calyx of Held presynaptic terminal followed by three-dimensional reconstruction to assess alterations in gross morphology following molecular perturbations (Montesinos et al., 2015; Radulovic et al., 2020; Keine et al., 2022). In combination with viral-vector delivery to perform targeted molecular perturbation at distinct developmental time points, this method allows for fast and cost-effective analysis of synaptic gross morphology of presynaptic terminals at different developmental stages. This protocol was developed with the mouse calyx of Held but it is applicable to other preparations and a wide range of model organisms, mammalian and invertebrate, where fluorescent labeling of the target structure can be achieved.

## Materials and reagents

**Biological materials**

1. Laboratory mice (Rac1^tm1Djk^/J, P28, either sex) (Jackson Laboratory, catalog number: 005550)

**Reagents**

1. Sodium phosphate monobasic (Sigma-Aldrich, catalog number: S0751)
2. Sodium phosphate dibasic (Millipore, catalog number: 567550)
3. 2,2,2-Tribromoethanol (Sigma-Aldrich, catalog number: T48402)
4. 2-Methyl-2-butanol (Sigma-Aldrich, catalog number: 152463)
5. Paraformaldehyde (Sigma-Aldrich, catalog number: 158127)
6. Sodium hydroxide solution 1 N (Sigma-Aldrich, catalog number: S2770)
7. Hydrochloric acid solution 1 N (Sigma-Aldrich, catalog number: H9892)

**Solutions**

1. Phosphate buffer (PB) 0.1 M (see Recipes)
2. Phosphate buffer (PB) 0.5 M (see Recipes)
3. Fixative solution (4% paraformaldehyde in 0.1 M PB, pH 7.4) (see Recipes)
4. Avertin (see Recipes)

**Recipes**

1. **Phosphate buffer (PB) 0.1 M**
  **Table:** 

Reagent	Final concentration	Quantity
Sodium phosphate monobasic (solution A)	0.1 M	0.6 g in 50 mL of ultrapure H_2_O
Sodium phosphate dibasic (solution B)	0.1 M	2.4 g in 170 mL of ultrapure H_2_O
Total	0.1 M	220 mL
  - Prepare 50 mL of 0.1 M sodium phosphate monobasic solution by dissolving 0.6 g of sodium phosphate monobasic (MW = 119.98 g/mol) in 50 mL of ultrapure water (solution A) under constant stirring.
  - Prepare 170 mL of 0.1 M sodium phosphate dibasic solution by dissolving 2.4 g of sodium phosphate dibasic (MW = 141.96 g/mol) in 170 mL of ultrapure water (solution B) under constant stirring.
  - Slowly pour solution A into solution B while stirring and monitoring pH.
  - Stop when pH settles at 7.3–7.4.
  - Filter with a sterile bottle-top filter and store at 4 °C until ready to use for up to one month.
2. **Phosphate buffer (PB) 0.5 M**
  **Table:** 

Reagent	Final concentration	Quantity
Sodium phosphate monobasic (solution A)	0.5 M	3 g in 50 mL of ultrapure H_2_O
Sodium phosphate dibasic (solution B)	0.5 M	14 g in 170 mL of ultrapure H_2_O
Total	0.5 M	220 mL
  - Prepare 50 mL of 0.5 M sodium phosphate monobasic solution by dissolving 3 g of sodium phosphate monobasic (MW = 119.98 g/mol) in 50 mL of ultrapure water (solution A) under constant stirring.
  - Prepare 170 mL of 0.5 M sodium phosphate dibasic solution by dissolving 12 g of sodium phosphate dibasic (MW = 141.96 g/mol) in 170 mL of ultrapure water (solution B) under constant stirring.
  - Slowly pour solution A into solution B while stirring and monitoring pH.
  - Stop when pH settles at 7.3–7.4.
  - Filter with a sterile bottle-top filter and store at 4 °C until ready to use for up to one month.
3. **Fixative solution (4% paraformaldehyde in 0.1 M PB, pH 7.4)**
  **Table:** 

Reagent	Final concentration	Quantity
Paraformaldehyde	4%	4 g
Ultrapure H_2_O	n/a	100 mL
Total	n/a	100 mL
  - The process of preparing PFA solution should take place in a chemical fume hood.
  - Measure 70 mL of ultrapure water and heat to 55–60 °C while stirring on a hotplate stirrer. Avoid heating the solution above 65 °C, as this will degrade PFA.
  - Slowly add 4 g of paraformaldehyde.
  - Let stir and keep adding 1 N NaOH drop by drop until the paraformaldehyde is dissolved and the solution is clear. Let solution cool down.
  - Add 20 mL of 0.5 M PB and bring the final volume to 100 mL.
  - Adjust pH to 7.4 with 1 N HCl and 1 N NaOH.
  - Filter with a sterile bottle-top filter and store at 4 °C for up to one week or in 50 mL aliquots at -20 °C for up to one month.
4. **Avertin**
  **Table:** 

Reagent	Final concentration	Quantity
2,2,2-Tribromoethanol	44 mM	250 mg
2-methyl-2-butanol	n/a	0.5 mL
Ultrapure H_2_O	n/a	19.5 mL
Total	n/a	20 mL
  - Dissolve 250 mg of 2,2,2-Tribromoethanol into 0.5 mL of 2-methyl-2-butanol at 40 °C and while stirring. Make sure not to exceed 40 °C or the solution might degrade.
  - Add ultrapure water to a final volume of 20 mL, filter with a sterile syringe filter, and store in 0.5 mL aliquots at -20 °C for up to one year. Protect from light by using amber/brown tubes and store in a separate light-protected box. Do not refreeze unused Avertin as it might reduce the anesthesiologic effect. Discard the solution and prepare fresh if any of the following conditions occur:
    i. Expiration date (one year) exceeded.
    ii. Solution has turned yellow in color.
    iii. Solution started to crystallize.

**Laboratory supplies**

1. Magnetic stirring bars (VWR, catalog number: 442-0368)
2. Bottle-top filter (Thermo Fisher, Nalgene RapidFlow, catalog number: 596-3320)
3. Syringe filter (Millipore Millex, 0.22 μm, catalog number: SLGPR33RS)
4. Single-use hypodermic needles (Braun Sterican, 25G, 25 mm)
5. Three-way stop cock (GPC Medical, catalog number: DIS122)
6. Forceps (Fine Science Tools, Dumont #3, catalog number: 11231-30)
7. Extra Fine Bonn scissors (Fine Science Tools, catalog number: 14084-08)
8. Bonn scissors (Fine Science Tools, catalog number: 14184-09)
9. Surgical scissors (Fine Science Tools, catalog number: 14000-13)
10. 12-well plate (CytoOne, catalog number: CC7682-7512)
11. Aqua-Poly/Mount (Polysciences, catalog number: 18606-20)
12. Microscope slides (Fisher Scientific, Fisherbrand Superfrost Plus, catalog number: 22-037-246)
13. Cover glass (VWR, catalog number: 48404-453)
14. Razor blades (Procter & Gamble, Astra Superior Platinum Double Edge)
15. Superglue (3M, Scotch)
16. Modeling clay (VWR, catalog number: 470149-616)
17. Zirconia ceramic blades (Cadence Blades, catalog number: EF-INZ10)

## Equipment

1. Laboratory hotplate stirrer (VWR, catalog number: 442-1271)
2. Dissection tray (Fisher Scientific, Epredia Shandon, catalog number: 73092)
3. Mechanical pipette 0.5–10 μL (Eppendorf, Research Plus, catalog number: 3123000071)
4. Mechanical pipette 10–100 μL (Eppendorf, Research Plus, catalog number: 3123000047)
5. Digital pump (Ismatec, MS-4/12, catalog number: ISM597D)
6. Vibrating blade microtome (Leica Biosystems, VT1200 S, catalog number: 14048142066)
7. Confocal microscope (LSM 700, AxioObserver, Carl Zeiss Microscopy)

## Software

1. ZEN blue (Carl Zeiss Microscopy, v3, RRID:SCR_013672)
  - Commercial software
  - Minimum hardware requirements: 3 GHz Intel i5 quad-core CPU, 4 GB RAM, 32 bit graphics adapter with 4 GB RAM
2. Imaris Microscopy Image Analysis Software (Bitplane, Oxford Instruments, v10.0, RRID:SCR_007370)
  - Used with Imaris Measurement Pro feature
  - Commercial software
  - Minimum hardware requirements: 3 GHz dual core CPU (64-bit), 8 GB RAM, NVIDIA Quadro P400 Graphics Card with 2 GB RAM
  - Alternative software: Amira (RRID:SCR_007353)
  - Free alternative software: Fiji/ImageJ, RRID:SCR_002285)
3. MATLAB (MathWorks, v9.12, RRID:SCR_001622)
  - Commercial software
  - Minimum hardware requirements: Intel or AMD x86-64 processor, 4 GB RAM
  - Free alternative software: Python (RRID:SCR_008394), R (RRID:SCR_001905)

## Procedure

- **Transcardial perfusion**
*Note: A detailed protocol on transcardial perfusion including images and further methodological considerations can be found in Wu et al. (2021).*
  1. The transcardial perfusion should take place in a chemical fume hood.
  2. Prepare solutions (PB and 4% PFA), pour into glass beakers, and connect to pump. Prefill tubing with respective solutions and remove air bubbles. Switching between solutions (step A15) can be aided by using a three-way stop cock connected to PB, 4% PFA, and the injection needle.
  3. Weigh the animal.
  4. Deeply anesthetize the animal via intraperitoneal injection of Avertin (250 mg/kg body weight).
  5. Position the mouse in a supine position on a dissection tray and secure the limbs with clamps or laboratory tape.
  6. Locate the most caudal end of the sternum indicating the caudal end of the rib cage.
  7. Make a midline incision into the abdominal cavity through the skin and muscle layers just caudal of the rib cage.
  8. Open thorax cavity by cutting through the diaphragm, taking care not to damage the heart.
  9. Cut along the lateral wall on either side of the rib cage and lift it to expose the heart.
  10. Locate the left ventricle and right atrium. The right atrium has a characteristic dark red color. The left ventricle forms the tip of the heart.
  11. Insert a 25-gauge needle into the caudal portion of the left ventricle at a flat angle, being careful not to puncture the septum.
  12. Make a small incision in the right atrium with scissors so that rapid blood flow occurs.
  13. Turn on the pump to flush ice-cold 0.1 PB into the left ventricle at a flow rate of 1–2 mL/min and keep the needle in place using modeling clay or laboratory tape. The blood should be washed out through the right atrium.
  14. Continue to flush with PB until the mouse’s body weight has been flushed three times and the liquid leaving the right atrium is clear and free of blood (e.g., if the animal’s weight is 10 g, inject a total volume of 30 mL of PB). Examine the liver; it should change color from dark red to pale yellow or white.
  15. Inject fixative (4% PFA) using a three-way stop cock, making sure that the needle stays in place. Double-check tubing to make sure there are no air bubbles present. Organs and muscles should start to turn stiff after a few minutes.
  16. Allow the fixative to run until at least three times the mouse’s body weight has been injected (e.g., if the animal’s weight is 10 g, inject a total volume of 30 mL of fixative). Liver, limbs, and tail should then be stiff.
  17. Stop the pump and remove the needle.
  18. Decapitate the animal with surgical scissors.
  19. Carefully remove the brain from the skull, taking care not to touch the area of interest (e.g., cochlear nucleus or MNTB). Cut larger nerves with fine scissors instead of tearing them to minimize damage to the brain.
  20. Using forceps, carefully remove the meninges from the ventral surface of the brainstem.
  21. Immerse brain in 4% PFA in a 12-well plate at 4 °C overnight. The volume of PFA solution used should be at least three times the volume of the brain (e.g., > 1.5 mL for an adult mouse brain). Make sure the whole brain is fully submerged in the solution.
  22. After 12 h of post fixation, discard PFA and replace it with PB. The volume of PB solution used should be at least three times the volume of the brain (e.g., > 1.5 mL for an adult mouse brain). Make sure the whole brain is fully submerged in the solution. Keep the brain immersed in PB at 4 °C until further use. For best results, use the brain within a few hours after being placed in PB. Storing the brain in PB for several days might reduce fluorescence and impact imaging quality.
- **Slicing of fixed brain and mounting of brain slices**
  1. Remove the brain from PB and excise the rostral half of the cerebrum with razor blades.
  2. Using super glue, adhere the brain on the cut surface to the specimen holder. Handle the brain with caution to avoid touching the brainstem.
  3. Immerse the brain into PB and orient the specimen holder so that the ventral surface of the brainstem faces the vibrating microtome blade.
  4. Using the microtome, slice the brain at a thickness of 40 μm, moving the blade at a rate of 20–50 μm/s.
  5. Identify the region of interest and collect the slices containing this region in PB solution.
  6. Mount the slices on microscope slides and remove any excess PB. Allow the slices to dry almost completely; then, add one drop of Aqua-Poly/Mount to each slice, taking care to avoid the formation of air bubbles. Cover the slice with a cover glass.
  7. Allow Aqua-Poly/Mount to harden for 24 h at room temperature. Store completed samples at 4 °C in the dark when not in use. Properly prepared samples can be stored for several months and imaged multiple times.
- **Confocal imaging of presynaptic terminals**
  1. Locate the MNTB region containing EGFP-labeled calyx terminals using a low-magnification objective. Select calyx terminals that are well separated from neighboring terminals for subsequent reconstruction.
  2. Switch to a 63× oil immersion objective lens (Plan-Apochromat 63×/1.4 Oil DIC M27) and select an imaging area that contains multiple well-separated calyx terminals.
  3. Image the membrane-bound EGFP expressed in the cells using an excitation wavelength of 488 nm and an emission wavelength of 518 nm.
  4. Select the Scan Mode Frame and set the image size to 1,024 × 1,024 pixels.
  5. Set Speed to 8 and Averaging to 4 lines. Set bit depth to 16 bit and the scan mode to bidirectional.
  6. Set the pixel size to 0.1 μm × 0.1 μm × 0.35 μm.
  7. Optimize the microscopy image by adjusting the laser power, gain, and offset. Images suitable for 3D reconstruction will have low background noise, high contrast, and are not saturated (Figure 1). The fluorescence signal should be within the range of the detector. Use the range indicator to adjust laser power and gain. If saturated areas are detected, lower laser power and gain until there are no saturated pixels in the image.
    **Figure 1.**
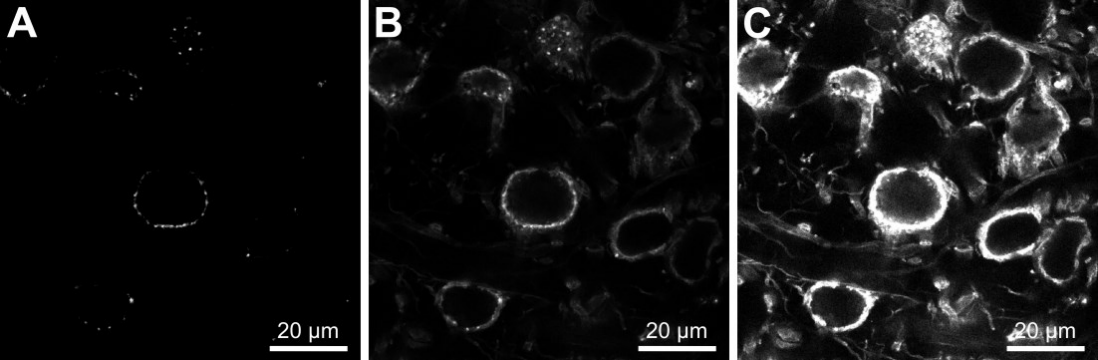
Adjusting laser power and signal gain to improve confocal images. When acquiring confocal images, the fluorescence signal should be within the range of the detector. (A) Signal too weak, structural details are lost due to low laser power and signal gain. (B) When laser power and signal gain are adjusted, structural details become visible and calyceal terminals can be imaged. (C) Setting laser power and signal gain too high will result in saturation of image details and loss of structural details. Images were obtained using a 63× oil immersion objective.
  8. Set the imaging depth in z-axis so that the whole presynaptic terminal and at least 5 μm on either end are within the imaging volume.
- **3D reconstruction of presynaptic terminals**
  1. Start Imaris, select *Arena*, and drag and drop the image file containing the acquired z-stack into *Arena.*
  2. Double-click on the file to convert it to IMS format.
  3. Double-click to open the IMS file.
  4. Inspect the image volume in *3D View* and locate a well-isolated calyx terminal that is fully captured within the image volume.
  5. Select *Edit* > *Crop 3D* and crop image volume to contain the selected calyx terminal. Make sure to crop the calyx as close as possible including 1 μm margins around the calyx volume. Then, click *OK.* The *3D View* should now contain the selected calyx terminal only.
  6. In the *Surpass tree* tab on the left, select *Add new Surfaces.*
  7. Select the newly created surface object. In the *Create* tab, select *Skip automatic creation, edit manually.*
  8. Click *Settings* and select *Render on Slicer.*
  9. Use the slider *Slice Position* in the *Draw* tab to select a slice in the middle of the image volume in which the calyx terminal is clearly visible.
  10. In the *Mode* tab under *Drawing Mode*, select *Magic wand*, click *Draw.*
  11. Using the mouse cursor, create line-abound surfaces with the same pixel intensity.
  12. When not in *Draw* mode, vertices of the detected surface can be re-positioned with the mouse while holding down the *T* button and deleted using the *D* button.
  13. Adjust the surface area carefully to properly represent the dimensions of the calyx terminal.
  14. When satisfied with the selection, select the next slice from the *Slice position* slider and repeat steps D9–D13. Repeat this process for all slices until the whole calyx terminal is selected (Figure 2).
    **Figure 2.**
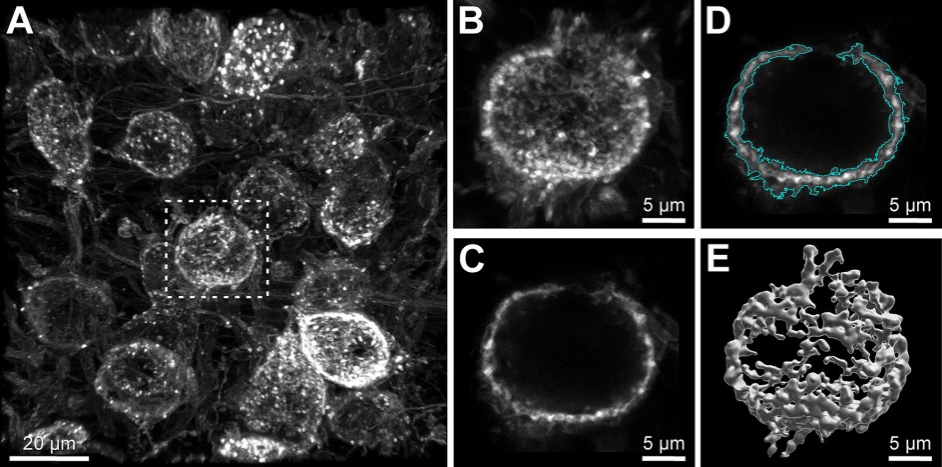
3D reconstruction of the calyx of Held presynaptic terminal. (A) Volumetric image containing mEGFP-labeled calyx terminals of a P28 mouse. Rectangle indicates the calyx terminal in B–E. Scale bar: 20 μm. (B) Volumetric image of single calyx terminal after three-dimensional cropping. Scale bar: 5 μm. (C) Single section through the middle of the calyx terminal shown in B. Scale bar: 5 μm. (D) Detection of surface borders (cyan) in the single section. Scale bar: 5 μm. (E) Full 3D reconstruction of fluorescent labeled calyx. Scale bar: 5 μm.
  15. Click *Create Surface* at the bottom of the *Draw* tab and turn off *Render on Slicer*.
  16. Using the tools in the *Edit* tab, the generated surface can be visually inspected and verified.
  17. If multiple surfaces have been created that should be merged into a single surface, select the surfaces to merge and click the *Unify* button located in the *Edit* tab.
  18. In the *Statistics* tab, select *Selection*. In the drop-down menu, select *Specific Values*. Then, select *Area* and *Volume* from the drop-down menu below. Data can be exported to CSV or XLS format using the *Export Statistics* buttons.

## Data analysis

Presynaptic surface area and volume were extracted from the 3D reconstructions and compared between treatment and control animals. To quantify the calyx’s overall shape, surface-area-to-volume ratios can be calculated by dividing the calyx’s surface area by its volume. Additional measures such as sphericity can be calculated and exported from Imaris but should be used with caution and depending on the expected shape of the structure of interest. Individual calyx terminals were considered independent samples, and calyx terminals that expressed only GFP served as control. Statistical analysis was conducted in MATLAB, but other statistical analysis software (e.g., R, GraphPad Prism, SPSS) can be used. Data distributions were tested for Gaussianity using the Shapiro-Wilk test (function swtest). Comparison of two groups was performed using a two-tailed unpaired Student’s *t*-test with Welch’s correction (normal distribution, function ttest2) or a two-tailed Mann-Whitney *U* test (non-normal distribution, function ranksum). Effect sizes were calculated using the MES toolbox in MATLAB (Hentschke and Stüttgen, 2011). Calyx terminals should be sampled from the same MNTB region in treatment and control mice to minimize bias introduced by location (Ford et al., 2009). The final dataset should contain a minimum of 15 calyces from at least three different animals per group, to minimize inter-individual differences. When using Cre-expressing viral vectors in combination with conditional knock-out mice to ablate synaptic proteins, animals were injected with viral vectors expressing either mEGFP (control) or both Cre recombinase and mEGFP (knock-out).

## Validation of protocol

This protocol has been validated independently by multiple researchers and results have been published in peer-reviewed publications (Montesinos et al., 2015; Radulovic et al., 2020; Keine et al., 2022). The procedure can be applied to different developmental stages and has been successfully applied in young, juvenile, and young adult mice. When using viral vectors to express Cre recombinase in combination with mEGFP to ablate proteins in conditional knock-out animal models, sufficient expression of Cre-recombinase should be validated by injecting the virus into a suitable Cre-reporter mouse line (e.g., B6.Cg-Gt(ROSA)26Sor^tm9(CAG-tdTomato)Hze^/J, RRID:IMSR_JAX:007909) with Cre-dependent expression of a fluorescent protein. To assess the effectivity and time course of Cre-mediated protein ablation, mRNA or protein levels can be quantified with qRT-PCR, Western plot, or immunohistochemistry in either cell cultures or brain slices.

## General notes and troubleshooting

**General notes**

All surgical procedures should be performed in accordance with a protocol approved by the respective Institutional Animal Care Use Committee. Slight variations in anesthesia, surgical procedures, pain management, and post-surgical care and follow-up may be required. Experiments have been performed in Rac1^tm1Djk^/J mice at age P28, but the protocol can be applied to other laboratory animals and developmental stages if mEGFP can be expressed in the target structure. However, large structures are more suited for this approach due to the resolution limit of confocal imaging. Subtle changes in cell morphology might go unnoticed using confocal imaging and require the use of super resolution or electron microscopy. All reagents are of molecular biology grade and should be stored according to the manufacturer’s recommendation. All solutions are prepared using ultrapure water (> 18 MΩ·cm at 25 °C) unless noted otherwise. For best results, solutions should be prepared daily. Solutions should be stored at 4 °C or -20 °C and can be used for one week or up to one month, respectively. Avoid multiple (> 3) freeze-thaw cycles.

**Troubleshooting**

**Table:** 

Problem observed	Possible reason	Solution
During perfusion, lungs expand, fluid outflow from nose/mouth	Heart septum pierced with needle	Withdraw needle and insert at flat angle
After perfusion, brain contains blood, liver does not turn pale	Ineffective washout of blood due to air bubbles in perfusion system	Remove air bubbles in the perfusion system
	Ineffective washout due to blood clotting	Reduce anesthesia dose to avoid cardiac arrest and minimize time between anesthesia and perfusion
After perfusion, blood is washed out, but brain remains soft	4% PFA solution not working properly	Make sure to fully dissolve PFA, avoid overheating (> 60 °C), and use fresh solution
Images oversaturated with no details visible	Laser power or gain too high during imaging	Lower laser power and gain, use range indicator
During reconstruction, the calyx borders cannot be determined properly	Overlap of passing axons or neighboring terminals	Use well-isolated calyx terminal for reconstruction
